# Distrustful Complacency and the COVID‐19 Vaccine: How Concern and Political Trust Interact to Affect Vaccine Hesitancy

**DOI:** 10.1111/pops.12871

**Published:** 2022-12-14

**Authors:** Fanny Lalot, Dominic Abrams, Maria S. Heering, Jacinta Babaian, Hilal Ozkececi, Linus Peitz, Kaya Davies Hayon, Jo Broadwood

**Affiliations:** ^1^ University of Kent; ^2^ University of Basel; ^3^ Belong—The Cohesion and Integration Network; ^4^ The Open University; ^5^ Belong—The Cohesion and Integration Network

**Keywords:** compliance, COVID‐19, political trust, vaccination intentions, vaccine hesitancy

## Abstract

We test the hypothesis that COVID‐19 vaccine hesitancy is attributable to *distrustful complacency*—an interactive combination of low concern and low trust. Across two studies, 9,695 respondents from different parts of Britain reported their level of concern about COVID‐19, trust in the UK government, and intention to accept or refuse the vaccine. Multilevel regression analysis, controlling for geographic area and relevant demographics, confirmed the predicted interactive effect of concern and trust. Across studies, respondents with *both* low trust and low concern were 10%–22% more vaccine hesitant than respondents with *either* high trust or high concern, and 26%–29% more hesitant than respondents with *both* high trust and high concern. Results hold equally among White, Black, and Muslim respondents, consistent with the view that regardless of mean‐level differences, a common process underlies vaccine hesitancy, underlining the importance of tackling distrustful complacency both generally and specifically among unvaccinated individuals and populations.

Since the outbreak of the coronavirus pandemic (COVID‐19) in 2020, substantial efforts have been directed toward the development of effective vaccines. The success of national vaccination campaigns is central to finally containing the virus and finding a way out of the pandemic. Yet, vaccine efficacy and safety are not enough to determine the success of these campaigns; vaccine acceptance among the general public is also key.

Different polls and studies conducted in 2020 revealed that a majority of the general public was willing to receive a COVID‐19 vaccine, although percentages varied across countries, groups, and particular localities (Sallam, 2021). However, a significant proportion remained “vaccine hesitant,” perhaps reinforced internationally by temporary halts in national rollouts while awaiting safety certification (as had substantially affected European Union countries; Leask, 2021) or by anti‐vaxxer misinformation campaigns (Loomba et al., 2021). In the United Kingdom, for example, research suggests that up to a third of people have been exposed to anti‐vaccine messages (Duffy, 2020). These and other factors might undermine a significant number of people's intentions to seek or accept the offer of being vaccinated and potentially compromise effort to reach herd immunity (Wiysonge et al., 2022). Therefore, it is vital to understand the psychology of vaccine hesitancy.

## Vaccine Hesitancy

Vaccine hesitancy is not a new phenomenon. Prior to the COVID‐19 pandemic, scholars and practitioners were studying factors underlying individuals’ and parents' unwillingness to accept vaccines for themselves or their children (Dubé et al., 2013). Research highlighted a number of determinants of vaccine hesitancy. The 3C model originally cited confidence, convenience, and complacency (MacDonald, 2015) and was later updated to integrate calculation and collective responsibility (5C model; Betsch et al., 2018). According to this model, vaccine uptake is greater when trust in the effectiveness and safety of vaccines (*confidence*), perceived risks of disease (low *complacency*), engagement in extensive information search (*calculation*), and awareness of social benefits (*collective responsibility*) are high, and structural barriers (*convenience*) are low (Betsch et al., 2018; Ryan & Malinga, 2021).

Vaccine hesitancy in the COVID‐19 pandemic may not be directly comparable to that in previous contexts. No virus in living memory has disrupted social lives and society as pervasively as COVID‐19. It is possible that the lockdowns and other restrictive measures adopted to stop the spread of the virus shifted individuals' calculus about receiving a vaccination when they considered additional consequences for economic activity, income, social life, and individual freedom. In fact, evidence suggests that COVID‐19 vaccine hesitancy is related to more general attitudes toward protective health behavior against the virus, such as respecting social distancing recommendations, taking regular tests, or wearing a face mask (Freeman et al., 2022). This suggests people might have similar considerations when accepting (or not) a COVID‐19 vaccine and adopting other protective measures in general.

Be that as it may, recent evidence on COVID‐19 vaccine hesitancy supports the role of 5C factors, predominantly confidence and complacency (Lazarus et al., 2022; Machingaidze & Wiysonge, 2021; Troiano & Nardi, 2021; Wiysonge et al., 2022). Collective responsibility (willingness to protect others) also drives vaccination intentions (Loomba et al., 2021). Convenience, for its part, has been less of an issue (in Western countries at least), as many governments ordered more than enough vaccine doses for their populations. Research has also identified specific effects of other factors such as conspiracy beliefs, social media use, and exposure to misinformation (Allington et al., 2021; Chadwick et al., 2021; Enea et al., 2022; Freeman et al., 2022; Loomba et al., 2021).

Crucially, most of the work on vaccine hesitancy considers the different underlying factors separately, looking for their unique or cumulative effects. In contrast, we argue that these factors might *interact* with each other, so that specific combinations might lead to distinctively low or high levels of vaccine hesitancy. Specifically, we build on past work investigating the psychological mechanisms of vaccine hesitancy, combined with recent evidence identifying an interactive effect of COVID‐19 concern and political trust on the adoption of protective health behavior (Lalot et al., 2022). We propose that the combination of low concern and low political trust (i.e., in the authorities responsible for the vaccination program) should characterize people with the most vaccine hesitancy. In the following sections, we describe the role of both variables, separately and in interaction.

## The Role of Concern

### Self‐Concern

People are generally more willing to adopt protective behavior when they are more concerned about the situation at stake—in the sense of considering the situation as more important, worrying, and directly involving them. This applies across a number of life domains, including organizational (Rundmo & Hale, 2003), environmental (Rhead et al., 2015), and, crucially, health behavior (Iversen & Rundmo, 2002). In the health domain, concern is generally considered as directly emanating from perceived personal risk, and the two variables are often strongly correlated (e.g., see Beebe‐Dimmer et al., 2004; Lipkus et al., 1999). Health studies also generally find health concern is predictive of the adoption of health behavior intended to detect or prevent the potential disease (see also Tamers et al., 2014). Similar evidence applies to vaccination intentions specifically (as per the complacency dimension of the 5C model described above; Betsch et al., 2018; Enea et al., 2022; Ryan & Malinga, 2021; Taylor‐Clark et al., 2005).

Some researchers have distinguished between objective and subjective risk level and concern. It seems reasonable to expect an association between a current level of objective epidemiological risk and subjective concern (Loewenstein & Mather, 1990). During the COVID‐19 pandemic, Nelson et al. (2020) found that COVID‐19 case rates were positively associated with COVID‐19 concern. Concern levels, in turn, were positively related to self‐quarantining behavior (see also Kleitman et al., 2021). However, there was no independent relationship between COVID‐19 case rates and self‐quarantining behavior. Other recent evidence showed that general concerns over the virus were associated with greater engagement in social distancing (Shook et al., 2020). Hence, it seems likely that the subjective and psychological evaluation of the situation is more focal than the objective risk in people's behavioral decisions.

### Concern and Anxiety in Politics

The relevance of concern for behavior in time of crisis is further supported by insights from political models of emotional processing (see Marcus, 2000), notably around anxiety. The theory of affective intelligence proposes that anxiety can arise when a surveillance system detects novel situations and potential threats (Marcus et al., 2000). Anxiety prompts individuals to actively search for new information to deal with the perceived threat and leads to political decisions that are based on this newly collected information rather than on political heuristics and partisanship, as would be the case in less anxious times (MacKuen et al., 2007). Consequently, anxiety can decrease partisanship biases in decision making. For example, a study testing affective intelligence theory's predictions found that anxiety (but not anger) about COVID‐19 was related to greater support for measures restricting civil liberties during the first wave of the pandemic in five European countries, above and beyond political partisanship (Vasilopoulos et al., 2022).

Other accounts of “anxious politics” similarly suggest that *unframed threats* such as public health crises (i.e., a widely agreed‐upon cause of harm involving a risk of imminent harm) cause anxiety that motivates greater information seeking related to the threat. This can increase support for protective policies—irrespective of political partisanship (Albertson & Gadarian, 2015). Similarly, recent work shows that partisans were not sensitive to the ostensible source of a COVID‐19 threat–related message, possibly indicating that they concentrated on the message rather than the source (Gadarian et al., 2021a). It may follow that when partisans do react differently to COVID‐19 (e.g., Democrats adopting more protective behavior than Republicans in the USA), this may reflect a differing perceptions of risk and resulting level of anxiety, rather than a different reaction to anxiety per se (Gadarian et al., 2021b).^1^

### Identity Fusion and Concern for Others

Crucially, concern often extends from mere personal concern to embrace larger social issues that may or may not impact the self directly (Abrams & Travaglino, 2018; Loewenstein & Mather, 1990). Indeed, people might also adopt protective behavior when they perceive the situation as concerning for others and not just themselves. Prior to this pandemic, vaccination intentions had been shown to increase as a function of *both* personal risk (i.e., self‐concern) and worry about protecting vulnerable others (i.e., other‐concern; Vietri et al., 2011), which is also reflected in the collective responsibility dimension of the 5C model. Moreover, although self‐concern and other‐concern are theoretically independent, they are also strongly intertwined—notably, when people feel more strongly connected or psychologically “fused” to wider social groups, such as in time of global crisis (Gómez et al., 2020). In such times, people make less distinction between the self and others and tend to perceive the situation as equally important or concerning for themselves and for others. Identity fusion is related to greater empathic concern (Landabur et al., 2022) and, in turn, to greater willingness to engage in self‐sacrifice and greater pro‐social behavior for the benefit of the community (Segal et al., 2018). In the context of the COVID‐19 pandemic, it could then be expected that self‐concern and other‐concern align (Abrams, Lalot, et al., 2021) and together drive the adoption of protective behavior for the sake of both the self and the community (see, e.g., Kleitman et al., 2021), including taking the vaccine (Enea et al., 2022).

Correspondingly, people who *feel* less concerned—both for themselves and for others—should be less motivated to receive the vaccine, potentially jeopardizing the collective endeavor to subdue the virus.

## The Role of Political Trust

### Political Trust and Partisanship

Regardless of their levels of concern, individuals may adopt protective behavior for other reasons—a key one of these being political trust. *Political trust* refers to the confidence people have in their government and the extent to which they see their government as trustworthy and competent (Levi & Stoker, 2000). It is an evaluative attitude held by a citizen toward their political system or agents, with several components contributing to the overall evaluation, notably, technical competence or success, ethical and fair conduct, and perceived congruency with citizens' best interests (Bertsou, 2019; Citrin & Stoker, 2018; Devine et al., 2020; PytlikZillig & Kimbrough, 2016). *Trust* can refer to numerous institutional contexts (e.g., in the present local governance or state governance, Congress), although research suggests that people tend to express consistent and intercorrelated (positive or negative) evaluations of the different actors, showing a “halo” of trust or distrust across contexts (PytlikZillig et al., 2016).

Unsurprisingly, there is typically a link between political trust and political partisanship, with trust usually being higher for citizens sharing a common identity (Tyler & Degoey, 1995) and a party affiliation with the leadership (e.g., Hooghe & Oser, 2017; Pew Research Center, 2010). However, political trust cannot be reduced to partisanship and a mere in‐ versus outgroup effect. Partisans can be disappointed with their government's performance or latest policy decisions and nonpartisans can still judge that their government acts in a competent or ethical manner even if they disagree about specific policy decisions (Citrin & Stoker, 2018). In addition, partisanship and trust seem to align more strongly in biparty systems that have stronger representations of party oppositions along the lines of “us versus them” (e.g., the USA). However, the impact of partisanship is much less straightforward in multiparty systems where different parties often agree on diverse specific issues (e.g., most European countries; for comparisons, see, e.g., Givens & Luedtke, 2005; Hix, 1999; Huddy et al., 2018). In sum, political trust is related to, but distinct from, political partisanship and differently predicts a range of political views and actions.

### Political Trust and Compliance

Importantly, political trust influences citizens' political *and* nonpolitical behavior (for reviews, see Citrin & Stoker, 2018; Levi & Stoker, 2000). Most central for our present purpose, political trust is positively related to willingness to comply with rules and regulations enacted by the authorities: Citizens are more likely to respect the decisions of political institutions that they perceive as legitimate and competent (Marien & Hooghe, 2011; Tyler, 2001, 2006).

This positive effect of trust on compliance has been identified with respect to health regulations specifically. For example, during the 2014 Ebola epidemic in Liberia, low trust in the government was related to lesser compliance with public health policies (Blair et al., 2017). Participants imagining a potential smallpox outbreak were more likely to oppose strong policies such as quarantine and mandated vaccination when they distrusted their government, particularly when they thought the government was likely to abuse powers, violate personal liberties, and treat certain social groups unfairly in the process (Taylor‐Clark et al., 2005). A narrative account of smallpox outbreaks in the late 19th and 20th centuries similarly revealed that a sense of distrust in the government, increased by a poor handling of the epidemic, led to noncompliance and riots, ultimately exacerbating the outbreak (Leavitt, 2003; see also Siegrist & Zingg, 2014).

Similar findings arose more recently with respect to the COVID‐19 pandemic. Several large‐scale international surveys found positive associations between individuals' levels of political trust and their adoption of health‐protective behavior (Han et al., 2021; Pagliaro et al., 2021; see also Devine et al., 2021, for an early review of the literature). Others have found the same association at the regional or country level (Bargain & Aminjonov, 2020; Kestilä‐Kekkonen et al., 2022; but see Woelfert & Kunst, 2020). Probably as a consequence of this increased compliance, higher trust was also associated with a lower excess mortality rate during the pandemic across 113 countries (Farzanegan & Hofmann, 2022). Interestingly, the potential influence of trust is especially apparent when citizens do not perceive the regulation as directly benefiting their self‐interest. For example, the positive effect of political trust on acceptance of redistributive policies is stronger among conservatives (ideologically less favorable to such measures) than among liberals (Rudolph & Evans, 2005). In this case, political trust may sustain compliance even when personal interest or concern is low. Put differently, when people's level of concern is low, political trust could mitigate low motivation to adopt protective behavior (see also Baum et al., 2009). This reasoning suggests that although the presence of either political trust or concern is necessary, and either should be sufficient to motivate vaccine uptake, the absence (or low levels) of both would substantially increase vaccine hesitancy.

## The Distrustful Complacency Hypothesis

Recent theorizing on *aversion amplification* (see Abrams & Travaglino, 2018) focused on the way political trust interacts with concern about a focal political issue to affect political intentions. The central principle is that certain intentions or behavioral tendencies become amplified when political trust combines interactively with other motivators, such as concern. For example, Abrams and Travaglino showed that the relationship between people's concern about immigration and their sense that this posed a threat (both symbolic and realistic) was moderated by their level of political trust. Specifically, high political trust served as a buffer for participants reporting high concern, leading to lower perceived threat than among participants with low political trust (Abrams & Travaglino, 2018). In a similar vein, Lalot et al. (2021) found that high political trust served as a buffer against uncertainty in the early days of the COVID‐19 pandemic, leading to less extreme levels of perceived threat from the virus among highly uncertain participants as compared with low political trust.

Based on this reasoning, Lalot et al. (2022) advanced a *distrustful complacency* hypothesis, which they tested on small opportunity samples of respondents (*N* = 400) in France and Italy during lockdown in spring 2020. The distrustful complacency hypothesis extrapolates the theoretical principles of the aversion amplification process to consider how political trust and concern affect compliance with governmental regulations (in this case, adoption of health‐protective behaviors against COVID‐19). The basic principle is that when particular levels of concern and trust *both* imply a common behavioral tendency (e.g., noncompliance), that tendency will be significantly amplified relative to when only one or neither implies that tendency. Political trust and concern are alternative routes that affect compliance: People might not follow government's regulations either because they are not worried about the consequences of noncompliance of because they do not trust the government. People who lack both concern and trust would therefore be markedly less motivated to comply. Consistent with this hypothesis, Lalot and colleagues found that respondents' intentions to comply with governmental COVID‐19 behavioral restrictions were significantly lower among those who had *both* lower levels of concern and lower political trust than among those who had either higher concern (regardless of trust) or higher trust (regardless of concern; Lalot et al., 2022).

Other researchers have conceptually replicated these findings: Seyd and Bu (2022) observed that trust in government and perceived health risk (or worry) interact to predict compliance across three countries and different time points, so that trust is a more important predictor of compliance at low levels of perceived risk, but its effect shrinks as individuals' perceptions of risk increase. Vasilopoulos et al. (2022) similarly found a positive effect of political trust on support for stringent measures in five European countries (phone surveillance and curfews), but only at low levels of anxiety. When participants expressed high anxiety, they endorsed the measures irrespective of their level of trust. In sum, there is accumulating evidence that trust and concern interact to predict the adoption of health‐protective behavior in the context of COVID‐19.

The aforementioned work echoes some themes in the literature of vaccine hesitancy, highlighting the importance of concern (i.e., complacency and collective responsibility) and trust (i.e., confidence) in the adoption of the target behavior. However, it also differs from it in two important respects: First, it focuses on political trust (in the government) rather than trust in the safety and efficacy of the vaccine or behavior itself. Second, it holds that there should be an interactive effect of concern and trust, whereas the 5C model considers them as separate, cumulative factors (Betsch et al., 2018; Ryan & Malinga, 2021). Therefore, it remains to be tested whether the distrustful complacency hypothesis is supported in the case of vaccination intentions and how well it can apply across a wider set of locations and groups.

### Is Distrustful Complacency Generalizable?

Demographers and researchers alike have pointed to higher levels of vaccine hesitancy among particular groups or communities, particularly noting lower vaccine take‐up among Black, Asian, and other minority ethnic groups within both the United Kingdom and the United States (Nguyen et al., 2021; Razai et al., 2021). However, what has not been addressed is whether the mitigation strategy should focus on different processes for different groups or whether it is a matter of degree whereby the same factors are influential. We suggest here that the psychological mechanisms underlying vaccine hesitancy should be largely common to all. Thus, the generalizability of distrustful complacency as a predictor of hesitancy has implications not just for the validity of theory but also for confidence about whether intervention strategies should adopt a similar approach across different groups.

If distrustful complacency is clearly linked to vaccine hesitancy, this suggests that a coherent psychological strategy selectively targeting trust and/or concern may be particularly effective in reducing hesitancy. This also has wider relevance to other health‐protective behaviors and the design of public information campaigns. The present research therefore provides a comprehensive test of the distrustful complacency hypothesis in two large studies.

### The Present Research

To summarize, we first expect main effects of both concern and political trust: People with lower concern should report greater vaccine hesitancy, and people with lower political trust should report greater vaccine hesitancy. Second, and more importantly, we also expect an interactive effect of political trust and concern, implying distinctively greater vaccine hesitancy when both trust and concern are at low levels (i.e., a state of distrustful complacency) than when at least one of them is high.

We (a) examine whether the hypothesis holds across multiple samples defined by different areas and by different ethnic composition within the UK (Study 1; over 8,500 respondents in Great Britain between December 2020 and February 2021), (b) replicate the test in a nationally representative sample (Study 2; over 1,000 British respondents in March 2021), (c) account for multiple demographic variables to see whether the psychological processes operate across these, and (d) are able to discern—by comparing the evidence from the two studies, the first conducted at the start of the vaccination program and the second at the midway point—whether the objective level of vaccine take‐up nationally changes or qualifies the impact of distrustful complacency.

## STUDY 1

### Method

#### Research Context and Data Collection

Study 1 is a cross‐sectional survey in which we measured concern about COVID‐19, political trust, and vaccine hesitancy. Data were collected as part of a large‐scale survey of social cohesion in the UK during COVID‐19 (Abrams, Broadwood, et al., 2021). The research project had a strong focus on the effect of place and aimed to compare the lived experiences of people from different areas during the pandemic. As such, it encompassed 13 different areas of residence in the UK (see details in the Electronic Supplementary Material, ESM 1). As a second overall aim, the project was interested in personal differences in investment in social cohesion; it therefore included a sample of community activists (currently volunteering for different charities) from across Great Britain (*n* = 582). In addition, to enable reliable assessment of hesitancy among specific minority groups, samples were boosted with Black respondents and Muslim respondents to achieve nonoverlapping quotas of *n* = 500 each from across Britain. This strategy, albeit hindering the overall representativeness of the sample, ensured we could reliably compare psychological mechanisms between different social groups.

Sample size was determined prior to data collection based on feasibility and available funding. Data were collected from December 4, 2020, to February 2, 2021 (to provide temporal context, a timeline of events is detailed in ESM 2). At the completion of data collection for Study 1, 19% of the adult population in the UK had received a first dose of the vaccine, although only 1% had received the second dose. The staged vaccine rollout also meant that appointments at that time were only available for older residents in care homes, their carers, frontline health and social care workers, clinically extremely vulnerable individuals, and all aged 70 and over. Everyone else had to wait to be called forward (BBC News, 2021).

#### Participants and Procedure

Respondents were recruited to complete an online survey through two complementary channels: via Qualtrics Panels and via social media and distribution by partnering local councils and charities.^2^ All respondents were remunerated for their participation (£5, equivalent to 7 USD). In both studies, all participants gave their informed consent prior to starting the survey. They were informed of their right to withdraw at any time as well as to retract their consent even after finalizing the study (up to 3 weeks following completion and through a unique anonymized code; no participant retracted their consent). The research received approval from the School of Psychology Ethics Committee at the University of Kent (ethics ID: 202015886922686497). A total of 8,743 respondents completed the survey. Participants who failed an attention check or completed the survey implausibly quickly were automatically excluded from the sample (*n* = 114; i.e., 1.3% exclusion rate), leaving 8,629 complete questionnaires. Our contractor, Qualtrics Panels, had determined the “reasonable” time completion for the survey based on an initial soft launch including about 100 participants. Median completion time for the survey was 32 min, and the speeding check was set to 12 min (i.e., less than half the median completion time). Participants were 3,814 men and 4,762 women (53 other or undisclosed) ranging from 18 to 91 years of age (*M* = 47.74, *SD* = 16.57). Most respondents self‐described as White or White British (77.6%), with smaller numbers from Asian (8.8%), Black (6.6%) or mixed (1.8%) ethnic background. All demographics are reported in ESM 1. A sensitivity power analysis revealed that the sample size was sufficient to detect a small interaction effect (*d* = .08) at 95% power (*α* = .05).

#### Materials

Some of the items were drawn verbatim or adapted from past research, whereas others were developed by the research team for this project. All measures are reported in Table 1 alongside the sources of reproduced and adapted items. Unless stated otherwise, all measures were assessed using 5‐point Likert scales.^3^

**Table 1:** Items, Scaling, and Reliability Indices for Measures of Concern, Trust, and Vaccine Hesitancy in Studies 1 and 2

Construct	Reliability (*α*/*ω*_*T*_)	Items	Scale	Source
*Study 1*				
Concern	–	(1) Compared with other things, how concerned are you about the impact of coronavirus for the UK?	1 = Not at all concerned, 5 = Extremely concerned	Adapted from Lalot et al. (2022)
Political trust	.73/.79	(1) Politicians are mainly in politics for their own benefit and not for the benefit of the community. (R)	1 = Strongly disagree, 5 = Strongly agree	Taken from Lalot et al. (2021)
		(2) Most members of the UK Parliament are honest.	1 = Strongly disagree, 5 = Strongly agree	Taken from Lalot et al. (2021)
		(3) I trust my local member of parliament to represent the interests of all communities across the constituency.	1 = Strongly disagree, 5 = Strongly agree	Adapted from Lalot et al. (2021)
		(4) I believe the UK Government is handling the causes and consequences of the pandemic competently.	1 = Strongly disagree, 5 = Strongly agree	Adapted from the COVIDistress Survey
		(5) I believe my local council (i.e., town or city or district) is handling the causes and consequences of the pandemic competently.	1 = Strongly disagree, 5 = Strongly agree	Self‐developed
Vaccine hesitancy	–	(1) Imagine that the NHS had plenty of supplies and contacted you next week to invite you to get vaccinated on a day of your choice in the next week. What would you decide?	1 = I would refuse without hesitation, 5 = I would accept without hesitation (6 = I have already received the vaccine, recoded for analyses as 5)	Self‐developed
*Study 2*				
Concern	.83/.85	(1) How concerned are you about consequences of the pandemic for you personally (such as your health, financial, or other aspects)?	1 = Not concerned at all, 7 = Extremely concerned	Self‐developed
		(2) How concerned are you about consequences of the pandemic for the people in your local area?	1 = Not concerned at all, 7 = Extremely concerned	Self‐developed
		(3) How concerned are you about consequences of the pandemic for the people in the UK in general?	1 = Not concerned at all, 7 = Extremely concerned	Self‐developed
Political trust	.92/.95	(1) Politicians are mainly in politics for their own benefit and not for the benefit of the community. (R)	1 = Strongly disagree, 5 = Strongly agree	Taken from Lalot et al. (2021)
		(2) Most members of the UK Parliament are honest.	1 = Strongly disagree, 5 = Strongly agree	Taken from Lalot et al. (2021)
		(3) How much trust do you have in the UK government?	1 = None at all, 5 = A great deal	Taken from the COVID‐19 Psychological Research Consortium Study
		(4) I believe the UK Government is handling the causes and consequences of the pandemic competently.	1 = Strongly disagree, 5 = Strongly agree	Adapted from the COVIDistress Survey
		(5) How well or badly do you think the UK Government is handling the issue of the coronavirus?	1 = Very badly, 5 = Very well	Taken from the Imperial College London YouGov Covid 19 Behavior Tracker
		(6) Over the next year, how much do you think Boris Johnson (Prime Minister and leader of the Conservative Party) can be trusted to handle the pandemic for the UK as a whole?	1 = Not at all, 5 = Completely	Self‐developed
		(7) Over the next year, how much do you think Boris Johnson (Prime Minister and leader of the Conservative Party) can be trusted to handle the pandemic for England?	1 = Not at all, 5 = Completely	Self‐developed
Vaccine hesitancy	.78/.78	(1) Imagine that the NHS contacted you next week to invite you to get vaccinated on a day of your choice in the next week. What would you decide?	1 = I would refuse without hesitation, 5 = I would accept without hesitation (6 = I have already received the vaccine, recoded for analyses as 5)	Self‐developed
		(2) How much do you trust that UK health authority (Medicines and Healthcare Regulatory Authority; MHRA) would only approve a new vaccine for distribution to the general public after proper tests ensuring the new vaccine is safe?	1 = Do not trust at all, 5 = Trust completely	Self‐developed

*Note*: *α* = Cronbach's alpha, *ω*_*T*_ = McDonald's omega total. (R) indicates reverse‐coded items.

*Sources*: COVIDistress Survey: an international research consortium with researchers from 50+ universities, led by Aarhus University (https://covidistress.com/covidistress‐2020/); COVID‐19 Psychological Research Consortium Study: Universities of Sheffield, Liverpool, Royal Holloway, and University College London, funded by ESRC (https://www.sheffield.ac.uk/psychology‐consortium‐covid19); Imperial College London YouGov COVID‐19 Behavior Tracker: a partnership between Imperial College London and YouGov (https://www.imperial.ac.uk/global‐health‐innovation/what‐we‐do/our‐response‐to‐covid‐19/covid‐19‐behaviour‐tracker/).

##### Concern

Concern was measured with a single item: “Compared with other things, how concerned are you about the impact of coronavirus for the UK?” (1 = *Not at all concerned*, 5 = *Extremely concerned*; *M* = 3.77, *SE* = .012).

##### Political Trust

Five items measured political trust: general (e.g., “Most members of the UK Parliament are honest”) and specific to COVID‐19 (e.g., “I believe the UK Government is handling the causes and consequences of the pandemic competently”; 1 = *Strongly disagree*, 5 = *Strongly agree*). Items loaded on a single factor and were aggregated into one average score, showing acceptable reliability (Cronbach's *α* = .73, McDonald's *ω*_*T*_ = .79; *M* = 2.70, *SE* = .008). A zero‐order correlation showed political trust and concern were only very weakly correlated, *r*(8,628) = .04, *p* < .001.

##### Vaccination Hesitancy

One item assessed hesitancy, addressing personal intentions to accept or refuse being vaccinated: “Imagine that the NHS [the UK National Health Service] had plenty of supplies and contacted you next week to invite you to get vaccinated on a day of your choice in the next week. What would you decide?” (1 = *I would refuse without hesitation*, 5 = *I would accept without hesitation*; *M* = 4.11, *SE* = .014). Some have made the case that single‐item measures are appropriate when used to assess a relatively clear construct, easily accessible by the respondent (see Nagy, 2002), which should be the case for vaccination intentions; some previous research has indeed successfully relied on such single‐item measures in the context of COVID‐19 (e.g., Faasse & Newby, 2020; Karlsson et al., 2021) as well as for other viruses (e.g., Jolley & Douglas, 2014; Nowak et al., 2020).

Overall, 14.8% of respondents said they would refuse the vaccine, 10.0% were unsure, and 73.6% would accept it. This is very much in line with the results of other national surveys at that time. For example, polls revealed that between 70% and 83% of UK respondents said they would take the vaccine (or had already received it) between December 14, 2020, and January 29, 2021 (YouGov, 2021). Respondents could also indicate that they had already received the vaccine (134 respondents or 1.6% ticked this option); we recoded this for analyses as the highest positive intention.

### Results

#### Analytical Strategy

Two strategies were implemented to account for potential differences between the different strata in the surveys and for effects of demographics. First, we conducted three‐level multilevel analyses with residential area as a random factor with two levels (nation, then county level as Levels 3 and 2, ICC = 0.011) and individual as Level 1. We initially ran both the constrained intermediate model (CIM; random intercept only) and the augmented intermediate model (AIM; random intercept and slope) and compared them. The likelihood‐ratio test showed that the AIM did not significantly improve the fit of the model, as compared to the CIM, *χ* ^2^(*df* = 6) = 1.16, *p* = .98. We therefore kept the simpler CIM model for further analyses. Second, we included the main demographics as covariates in the statistical model. We tested the model in two steps, first introducing the main predictors (concern and trust, both standardized, and their interaction) and then adding demographics as covariates (gender: −1 = male, 1 = female; ethnicity: −1 = non‐White, 1 = White; and age, socioeconomic status, and political orientation: all continuous and grand‐mean‐centered).^4^

#### Vaccine Hesitancy

**Figure 1:**
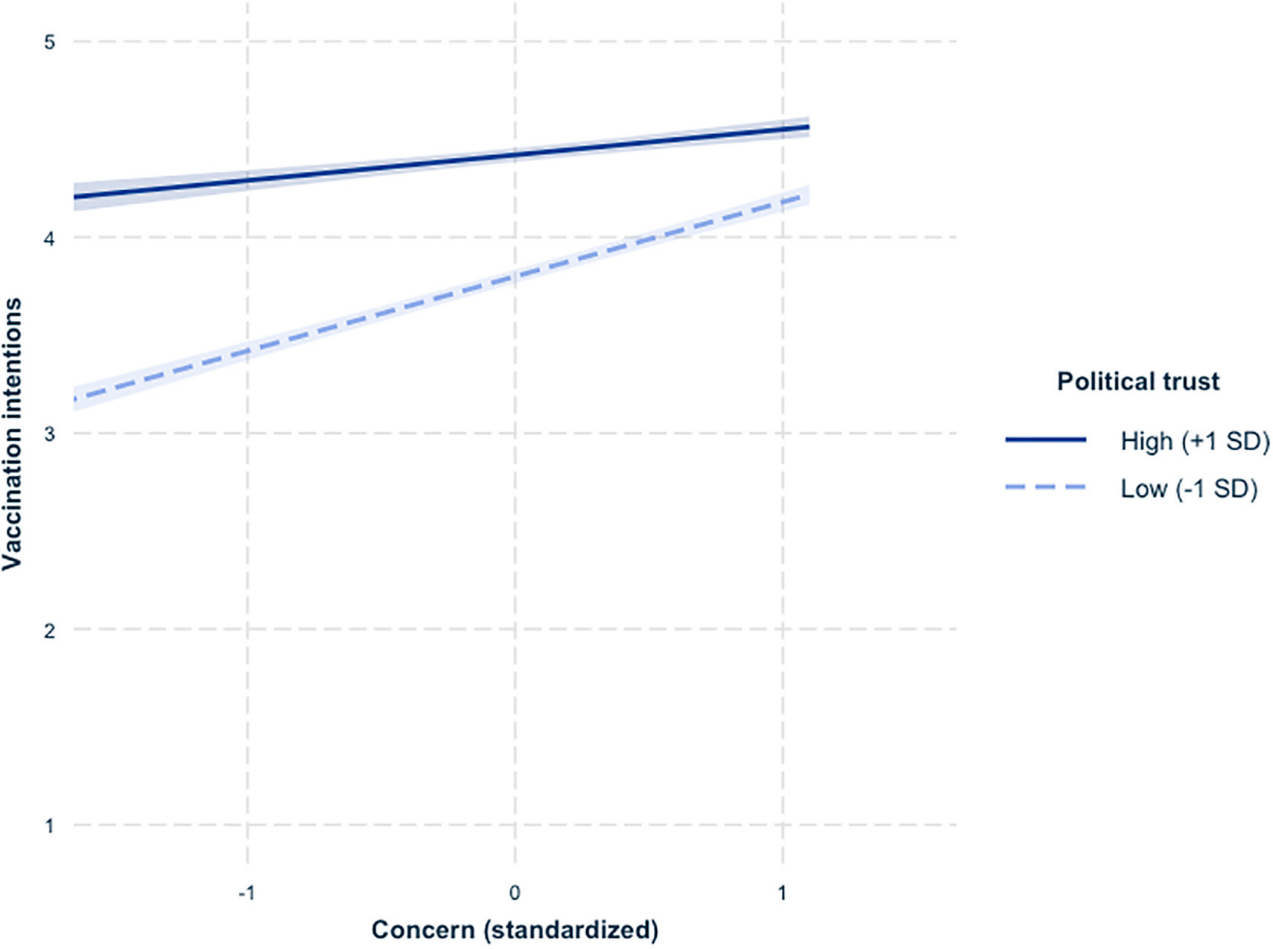
Vaccination intentions as a function of concern and political trust (higher scores represent greater vaccination intentions) in Study 1.

We regressed vaccine hesitancy on those predictors. Results, reported in Table 2, indicated main effects of both concern and political trust, showing that respondents with greater concern or greater political trust reported greater vaccination intentions. Of central interest, the expected Concern × Trust interaction was also significant and was unaffected by the introduction of covariates in the second step of the model (Figure 1).

**Table 2:** Summary of the Analyses Predicting Vaccination Intentions (Hierarchical Multilevel Linear Regression Model) in Study 1

	Step 1				Step 2			
	*b* (*SE*)	95% CI	*t*‐test	*p*‐value	*b* (*SE*)	95% CI	*t*‐test	*p*‐value
*Constant*	4.09 (.035)	[4.02, 4.16]	116.36	<.001	3.96 (.029)	[3.90, 4.02]	135.69	<.001
Concern	0.26 (.013)	[0.23, 0.28]	19.83	<.001	0.26 (.013)	[.23, .28]	20.16	<.001
Political trust	0.31 (.013)	[0.28, 0.33]	23.87	<.001	0.23 (.013)	[.20, .25]	17.15	<.001
Concern × Trust	−0.12 (.012)	[−0.15, −0.10]	−10.06	<.001	−0.11 (.012)	[−.14, −.09]	−9.56	<.001
Gender					−0.13 (.013)	[−.15, −.10]	−10.18	<.001
Age					0.21 (.014)	[.18, .24]	14.72	<.001
Ethnicity					0.27 (.017)	[.24, .31]	15.88	<.001
Socioeconomic status					0.11 (.013)	[.09, .14]	8.67	<.001
Political orientation					−0.11 (.012)	[−.16, −.10]	−9.56	<.001

*Note*: Positive coefficients indicate greater vaccination intentions (i.e., less vaccine hesitancy). Random effects are estimated for 101 counties (Level 2) embedded within the three nations of England, Scotland, and Wales (Level 3). Random effect of county: likelihood ratio test LRT(*df* = 1) = 0.26, *p* = .61. Random effect of nation: LRT(*df* = 1) = 0.00, *p* = 1.00. Step 1: *R* ^2^_adj Level 1_ = .118. Step 2: *R* ^2^_adj Level 1_ = .224.

Decomposition of simple effects revealed that among respondents with higher levels of concern (+1 *SD*), vaccination intentions were high overall, although they were even higher among respondents with higher political trust, *b* = .11, *SE* = .017, *t*(8,202) = 6.56, *p* < .001. Among respondents with lower concern (−1 *SD*), vaccination intentions strongly decreased when they also had lower levels of political trust, *b* = .34, *SE* = .018, *t*(8,193) = 18.94, *p* < .001. Put differently, among respondents who felt more distrustful (−1 *SD*), there was a strong link between concern and vaccination intentions, *b* = .37, *SE* = .016, *t*(8,197) = 22.89, *p* < .001, whereas this was reduced by more than half among respondents who felt more trustful (+1 *SD*), *b* = .14, *SE* = .018, *t*(8,199) = 7.82, *p* < .001. These results are consistent with our hypothesis that either high concern or high trust should be sufficient to induce greater vaccination intentions, but that intentions would be significantly lower if both concern and trust were low. Respondents with both low trust and low concern were 17% more vaccine hesitant (i.e., lower vaccination intentions) than respondents with low concern but high trust, 22% more hesitant than respondents with low trust but high concern, and 29% more hesitant than respondents with both high trust and high concern.

As random effects showed, when controlling for demographics, average vaccination intentions were similar between the different samples, with no significant differences across counties or across the three nations (see Table 2). The inclusion of demographic covariates in the second step of the model revealed that vaccination intentions were significantly higher among males, White people, older people, and wealthier people, as well as among those describing themselves as politically more left‐wing. The entire set of demographics explained an additional 10% of variance, where political trust and concern (and their interaction) had explained 12%.

#### Exploratory Analyses: Are There Specific Effects of Ethnicity and Religion?

During the pandemic a growing concern in the UK was the apparent reluctance of people from minority ethnic or religious backgrounds to accept the COVID‐19 vaccine. As of February 2021, National Health Service (NHS) England data revealed that White people were twice as likely to have received the vaccine than Black people, in the 80+ age group (Parveen & Barr, 2021). It has been argued that vaccine hesitancy within these communities could be due to targeted misleading claims such as the vaccine containing alcohol or pork (Humphreys et al., 2021) or “human guinea pigs” claims (Etutu & Goodman, 2021). Consistent with other findings, the present data reveal that non‐White respondents expressed greater vaccine hesitancy than White respondents. The central question then arises of whether ethnicity in any way changes the roles of political trust and concern in vaccine hesitancy. If the underlying psychological processes are the same, then ethnicity and religion should not moderate the interactive effect of trust and concern on hesitancy.

To test this, we conducted two additional multilevel linear regression analyses, the first examining effects involving Black ethnicity and the second examining effects involving Muslim faith. Both sets of analyses also controlled for age, gender, socioeconomic status, and political orientation, as well as the other religio‐ethnic factor, and added the two‐way and three‐way interaction terms between ethnicity/religion, political trust, and concern. These analyses were exploratory in nature, and we did not hypothesize a moderating effect of either ethnicity or religion.

Examining ethnicity first, the two‐way Political Trust × Concern interaction remained significant despite the introduction of other interaction terms, *b* = −.11, *SE* = .015, *t*(8,185) = −7.11, *p* < .001, and ethnicity (coded as White vs. non‐White) did not qualify the two‐way interaction; Political Trust × Concern × Ethnicity: *b* = −.02, *SE* = .015, *t*(8,184) = −0.12, *p* = .91. Similar results arose when considering Black versus non‐Black respondents (instead of White vs. non‐White): There was a significant main effect of ethnicity showing weaker vaccination intentions among Black respondents, *b* = −.40, *SE* = .026, *t*(7,995) = −15.42, *p* < .001. Most important however is the persistence of the significant two‐way Political Trust × Concern interaction despite the introduction of other interaction terms, *b* = −.09, *SE* = .025, *t*(8,194) = −3.68, *p* < .001, and Black ethnicity did not qualify the two‐way interaction; Political Trust × Concern × Ethnicity: *b* = .02, *SE* = .025, *t*(8,193) = 0.94, *p* = .35.

Finally, the analyses involving minority religious affiliation (i.e., Muslim faith vs. other religions) produced very similar results. There was a significant main effect of faith showing weaker vaccination intentions among Muslim respondents, *b* = −.19, *SE* = .027, *t*(7,658) = −7.15, *p* < .001. However, the two‐way Political Trust × Concern interaction remained significant despite the introduction of other interaction terms, *b* = −.10, *SE* = .023, *t*(8,125) = −4.32, *p* < .001, and religion did not qualify the two‐way interaction; Political Trust × Concern × Religion: *b* = .01, *SE* = .023, *t*(8124) = 0.52, *p* = .61. In other words, despite a difference in mean levels of hesitancy between majority and minority ethnicity or religion respondents, the psychological mechanisms underlying vaccine intentions are the same across these different ethno‐religious characteristics.

### Discussion

Study 1, conducted at a time when UK vaccination levels were low and the program was at an early stage, showed clear evidence of a distrustful complacency effect. Participants with both low concern and low political trust reported markedly higher vaccine hesitancy than participants with either high concern, high trust, or both.

For two reasons it seemed possible that this effect might change once a larger proportion of the population had received a first dose of the vaccine. On the one hand, after the most at‐risk age groups had been vaccinated, general levels of concern might decline, which would increase the proportion of people who are complacent and thus sustain or increase vaccine hesitancy. On the other hand, because there is a stronger descriptive norm of accepting a vaccine, perhaps trust levels would increase and thereby reduce the proportion who are distrustful. Even so, in both cases we still expected those who are most hesitant to be those who are distrustfully complacent—but this remained to be tested.

In addition, some methodological features of Study 1 limited its generalizability. Despite the large sample size and robustness of the results to the inclusion of different demographics, the study relied on single‐item measures for both concern and vaccine hesitancy (operationalized only as intention). The focus of the overall research project on place and individual experiences also meant we had to rely on a non‐probability sampling strategy, which limits the generalizability of the findings. Relatedly, the decision to include a larger number of minority group members also reduced the overall representativeness of the sample. To address these possible measurement and sampling limitations, and to extend the findings to an altered social context, we conducted a second study.

## STUDY 2

Study 2 served as a conceptual replication of Study 1 while also employing multi‐item constructs for political trust, concern about COVID‐19, and vaccine hesitancy, and relying on a nationally representative sample. Study 2 was conducted in March 2021 (all data were collected on March 5; see continued timeline in ESM 2), at which point 41% of the adult population had received a first dose of the vaccine but only 2% were fully vaccinated. The study design, measures, and sample size were preregistered (https://aspredicted.org/9xw2w.pdf). As described in the preregistration, other measures of compliance with COVID‐19 restrictions were included in the study, but they are not discussed in the present article.

### Method

#### Participants and Procedure

Respondents were recruited through the online platform Prolific Academic, specifying a representative sample of approximately 1,000 British respondents (stratified across sex, age, and ethnicity). We slightly oversampled to ensure that the final *N* would match this targeted sample size. A total of 1,074 respondents completed the survey. Eight participants who failed an attention check were excluded from the sample (0.7% exclusion rate), leaving 1,066 complete questionnaires (in this study, no exclusion was based on completion time, as no participants completed the survey in less than half the median completion time of 7 min). Sample characteristics were 520 men and 542 women (4 undisclosed) ranging from 18 to 83 years of age (*M* = 46.94, *SD* = 15.57). Most respondents self‐described as White or White British (85.0%), with smaller numbers from Asian (7.7%), Black (3.6%), or mixed (1.9%) ethnic background. All demographics are reported in ESM 5. A sensitivity power analysis revealed that the sample size was sufficient to detect a small interaction effect (*d* = .22) at 95% power (*α* = .05).

#### Materials

##### Concern

All items are reported in Table 1. Three items assessed concern about the pandemic, both for the individual and their larger group: “How concerned are you about consequences of the pandemic: for you personally (such as your health, financial, or other aspects); for the people in your local area; for the people in the UK in general?” (1 = *Not concerned at all*, 7 = *Extremely concerned*). Items loaded on a single factor and were aggregated into one average score, showing good reliability (Cronbach's *α* = .83, McDonald's *ω*_*T*_ = .85; *M* = 4.70, *SE* = .039). In line with theories of identity fusion and concern in time of crisis, it is worth noting that the items for self‐concern and other‐concern were strongly correlated (*r* ranging from .51 to .75). Additional tests yielded similar results when considering the aggregated three‐item measure or each item separately (in interaction with political trust), which further suggests it is appropriate to consider both self‐concern and other‐concern together in the present context.

##### Political Trust

Seven items measured political trust: general (e.g., “Most members of the UK Parliament are honest”), specific to COVID‐19 (e.g., “I believe the UK Government is handling the causes and consequences of the pandemic competently”), and relative to Prime Minister Boris Johnson (e.g., “Over the next year, how much do you think Boris Johnson can be trusted to handle the pandemic for the UK as a whole?”; 1 = *Strongly disagree*, 5 = *Strongly agree*). Items loaded on a single factor and were aggregated into one average score, showing good reliability (Cronbach's *α* = .92, McDonald's *ω*_*T*_ = .95; *M* = 2.46, *SE* = .029). A zero‐order correlation showed no significant relation between political trust and concern, *r*(1,065) = −.04, *p* = .15.

##### Vaccine Hesitancy

Two items measured vaccine hesitancy. The first item assessed intentions to accept or refuse the vaccine, just as in Study 1. Overall, 6.1% of respondents said they would refuse the vaccine, 4.5% were unsure, and 56.4% would accept it, while 32.9% had already received a first dose. As in Study 1, this latter option was recoded for analyses as the highest positive intention. A second item assessed a more general perception of the safety of the new vaccine (see, e.g., Danchin et al., 2018; Gerend et al., 2009): “How much do you trust that UK health authority would only approve a new vaccine for distribution to the general public after proper tests ensuring the new vaccine is safe?” (1 = *Not at all*, 5 = *Completely*). Both were aggregated into a single score of vaccine hesitancy, with higher scores representing higher acceptance of the vaccine (Cronbach's *α* = .78, McDonald's *ω*_*T*_ = .78; *M* = 4.26, *SE* = .028).

### Results

#### Measurement

We first conducted a factor analysis to ensure that the different items used were indeed measuring separate constructs. All items (three concern, seven political trust, and two vaccine hesitancy items) were entered in an exploratory factor analysis with Varimax rotation. Informed by the parallel analysis method and Velicer's MAP, extraction was fixed on three factors. The solution accounted for 66% of the variance and found all items to load on the expected factor with loadings greater than .64 (with the exception of two trust items with loadings of .41 and .52, respectively). No cross‐loading exceeded .32.

#### Vaccine Hesitancy

As in Study 1, we regressed vaccine hesitancy on political trust and concern (both standardized), and their interaction, and controlled for relevant demographics (age, gender, ethnicity, socioeconomic status, and political orientation). We relied again on multilevel modeling (county/nation) to account for the geographical dispersion of participants across Great Britain (see Table 3). It should be noted that the preregistration only mentioned linear regression models, and not multilevel analyses. However, it later seemed important to conduct the multilevel tests to ensure comparability with Study 1. It can be noted that simple linear regression models yielded similar results.

**Table 3:** Summary of the Analyses Predicting Vaccination Intentions (Hierarchical Multilevel Linear Regression Model) in Study 2

	Step 1				Step 2			
	*b* (*SE*)	95% CI	*t*‐test	*p*‐value	*b* (*SE*)	95% CI	*t*‐test	*p*‐value
*Constant*	4.27 (.030)	[4.21, 4.33]	143.93	<.001	4.11 (.036)	[4.04, 4.18]	113.50	<.001
Concern	0.11 (.026)	[0.06, 0.16]	4.19	<.001	0.12 (.025)	[0.07, 0.17]	4.65	<.001
Political trust	0.27 (.026)	[0.22, 0.32]	10.17	<.001	0.35 (.030)	[0.29, 0.41]	11.84	<.001
Concern × Trust	−0.08 (.024)	[−0.12, −0.03]	−3.22	.001	−0.08 (.023)	[−0.12, −0.03]	−3.35	<.001
Gender					−0.05 (.025)	[−0.10, 0.004]	−1.80	.072
Age					0.05 (.027)	[−0.01, 0.10]	1.76	.079
Ethnicity					0.22 (.037)	[0.15, 0.29]	6.03	<.001
Socioeconomic status					0.15 (.025)	[0.10, 0.20]	5.83	<.001
Political orientation					−0.21 (.030)	[−0.27, −0.15]	−7.04	<.001

*Note*: Positive coefficients indicate greater vaccination intentions (i.e., less vaccine hesitancy). Random effects are estimated for 95 counties (Level 2) embedded within the three nations of England, Scotland, and Wales (Level 3). Random effect of county (Step 2 model): likelihood ratio test LRT(*df* = 1) = 0.01, *p* = .94. Random effect of nation: LRT(*df* = 1) = 0.00, *p* = 1.00. Step 1: *R* ^2^_adj Level 1_ = .105. Step 2: *R* ^2^_adj Level 1_ = .197.

**Figure 2:**
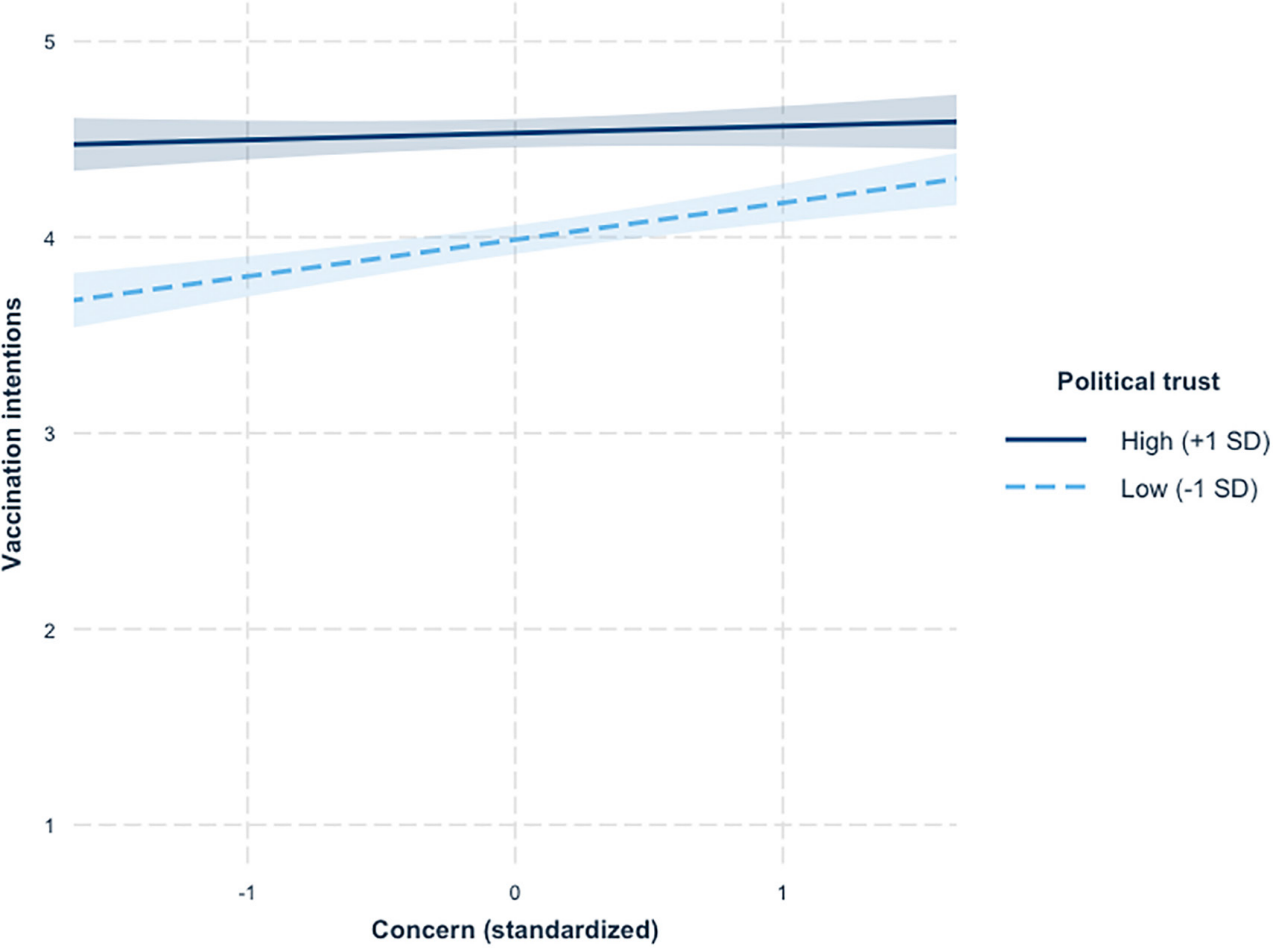
Vaccination intentions as a function of concern and political trust (higher scores represent greater vaccination intentions) in Study 2.

Consistent with the results of Study 1, the model yielded significant main effects of both concern and political trust, showing that respondents with greater concern or greater political trust reported greater vaccination intentions. More importantly, the expected Concern × Trust interaction was also significant and was unaffected by the introduction of covariates in the second step of the model (Figure 2).

Decomposition of simple effects revealed that among respondents with higher levels of concern (+1 *SD*), vaccination intentions were high overall, although they were still higher among respondents with higher political trust, *b* = .27, *SE* = .036, *t*(1,047) = 7.54, *p* < .001. Among respondents with lower concern (−1 *SD*), vaccination intentions strongly decreased when they also had lower levels of political trust, *b* = .43, *SE* = .038, *t*(1,048) = 11.20, *p* < .001. Put differently, among respondents who felt more distrustful (−1 *SD*), there was a strong link between concern and vaccination intentions, *b* = .19, *SE* = .034, *t*(1,046) = 5.75, *p* < .001, which disappeared among respondents who felt more trustful (+1 *SD*), *b* = .04, *SE* = .034, *t*(1,046) = 1.24, *p* = .21. These results again supported our hypothesis that either high concern or high trust should be sufficient to induce higher vaccination acceptance, but that hesitancy would be significantly greater if both concern and trust were low. Respondents with both low trust and low concern were 10% more vaccine hesitant than respondents with high concern, 19% more hesitant than respondents with high trust, and 26% more hesitant than respondents with both high trust and high concern.

The inclusion of demographic covariates in the second step of the model revealed that vaccination intentions were significantly higher among White respondents and those with higher socioeconomic status, as well as among those describing themselves as politically more left‐wing. Very consistent with the first study, the set of demographics explained an additional 9% of the variance, where political trust and concern (and their interaction) had explained 11%.

Analyses conducted on each item of vaccine hesitancy separately yielded very consistent results. The Concern × Trust interaction was significant in both instances. Finally, although the factor analysis suggested a unifactorial structure for each of the constructs, we probed for different effects of subdimensions of concern and trust. We repeated the analysis for each combination of 4 forms of trust (trust in institutions vs. trust in COVID‐19 response vs. trust in the prime minister vs. aggregated index) × 3 forms of concern (self‐concern vs. other‐concern vs. aggregated index). With the exception of one combination (trust in COVID‐19 response × self‐concern), all 11 interaction terms were significant, revealing considerable reliability of the findings above and beyond measurement specificities (see ESM 6 for more detailed results).

### Discussion

#### The Distrustful Complacency Hypothesis

As the world gradually emerges from the pandemic phase of COVID‐19, it seems clear that continued and repeated widespread vaccination is key for suppressing the virus and for societal recovery. Yet, vaccine hesitancy is a persistent challenge (Mutombo et al., 2022), not only for COVID‐19 but other viruses as well (Dubé et al., 2021). Thus, it remains crucial to understand the psychological mechanisms and profiles associated with vaccine hesitancy.

We hypothesized that concern and political trust are two routes that can interactively affect vaccine hesitancy. Specifically, either concern or political trust should be sufficient for vaccine acceptance, as the presence of one can compensate for the absence of the other. The absence of both, however, (i.e., distrustful complacency) would result in greater vaccine hesitancy.

This hypothesis was supported in both studies. Study 1 involved over 8,000 people in 13 different parts of the UK, including large numbers of often underrepresented minority groups (Black people and Muslim people). Study 2 reinforced the finding two months later with a UK representative sample, even though a larger proportion of the UK population had received a first dose of vaccination. Respondents low in both trust and concern were between 10% and 22% more hesitant about the vaccine than respondents with either high trust or high concern, and 26% to 29% more hesitant than respondents with both high trust and high concern. Some people accept the vaccine because they are highly concerned about the consequences of the pandemic (for themselves and for others). Others do so because they trust the political institutions responsible for enacting the vaccination program. However, those who, for whatever reasons, do not trust these institutions *and* are also not concerned about the virus are much likelier to be hesitant about vaccination.

As such, the present findings are consistent with a growing literature on vaccine hesitancy that identifies a positive role of several psychological factors and, notably, trust (or confidence) and concern (or complacency), both prior to (Betsch et al., 2018; Ryan & Malinga, 2021) and during the COVID‐19 pandemic (Loomba et al., 2021; Troiano & Nardi, 2021; Wiysonge et al., 2022). Our analysis also advances the state of this literature by testing the effect of concern and trust in interaction rather than as separate, additive factors. In parallel, the present findings also reinforce the validity of the distrustful complacency hypothesis, which in the context of the pandemic had so far only been tested with respect to compliance with (Lalot et al., 2022; Seyd & Bu, 2022) and support for restrictive government measures (Vasilopoulos et al., 2022).

Vaccine hesitancy was lower among male, left‐wing‐oriented, older, and wealthier respondents, and among White respondents (for similar findings, see, e.g., Lazarus et al., 2022; Schwarzinger et al., 2021). Media and practical attention have been paid to higher levels of vaccine hesitancy among particular ethnic categories, perhaps due to the spread of misleading information among them (Etutu & Goodman, 2021). However, we find that psychological variables explain at least as much variance as demographic factors. Moreover, the interactive relationship between concern and trust showed that there is a unifying psychological process beyond these demographics—distrustful complacency—that similarly affects White, Black, and Muslim respondents. Importantly, unlike demographics, concern and trust are subject to external influence and could therefore be targeted in policies and persuasion campaigns aiming to tackle vaccine hesitancy.

The strengths of the present research include the large sample sizes in both studies and the ability to examine both place‐based and demographic factors, as well as the timely periods of data collection. The inclusion of sufficiently large numbers of both Black and Muslim respondents enabled us to properly distinguish demographic and psychological factors of vaccine hesitancy. This important feature enhances our confidence that distrustful complacency is likely to generalize across different ethnic categories and across different‐sized communities, regions, and countries within Britain. Levels of concern and trust do differ by location. For example, trust in the UK government is notably lower within Scotland than within England (McDonnell, 2020), and levels of concern might differ locally depending on local infection rates; but the key point is that these contextual differences do not qualify the role of trust and concern as key motivators in vaccine hesitancy.

### Limitations and Constraints on Generality

Despite the strengths highlighted above, we acknowledge that the sampling strategies for each study constrain its generalizability. Study 1 was part of a large research project focusing on the role of place and individual experiences, which led us to target respondents from specific localities in Great Britain or with specific characteristics (i.e., subsamples of community activists, of Black people, and of Muslim people)—making the sample as a whole relatively nonrepresentative of the general population. Study 2 used a nationally representative sample, which addresses most of these limitations. However, because participants were recruited through an Internet‐based polling company, the sample is limited to opt‐in internet users with an interest for surveys (i.e., non‐probability sampling). Although respondents did not know the topic of the survey before provisional agreement to complete it, they might have been more interested in politics and societal topics in general than the general public (see Albertson & Gadarian, 2015, p. 140).

Notwithstanding these caveats, it is important to stress that our focus was on testing the consistency of hypothesized relationships between variables rather than establishing accurate national population estimates of mean‐level responses on individual variables. Nothing in the present evidence suggests that either demographic differences or at least two major ethno‐cultural factors would qualify the conclusions. Further work is needed to evaluate whether the same processes apply in nations other than the UK. However, taken together with Lalot and colleagues' (2022) evidence from France and Italy; Seyd and Bu's (2022) evidence from Austria, Germany, and the UK; and Vasilopoulos and colleagues' (2022) evidence from these same five countries, we have some confidence that our findings have much wider generality at least to Western societies.

Some measurement limitations must also be acknowledged. First, we only assess vaccination intentions with one (Study 1) or two items (Study 2), which limits our confidence in the reliability of the measure. Other researchers have used single‐item measures of vaccination intentions (see, e.g., Faasse & Newby, 2020; Karlsson et al., 2021; Nowak et al., 2020), but we recognize that more comprehensive instruments enable researchers to obtain both a more precise and more complex picture (see, e.g., Freeman et al., 2022; Shapiro et al., 2018).

It would also be valuable for future studies to examine the elements of concern and trust in greater detail. For example, self‐concern and concern for others were closely related in the present context but could theoretically constitute slightly different routes to compliance (Vietri et al., 2011). Likewise, it may also be useful to examine different aspects of trust (e.g., in the government in general, in leadership, in the health institutions) and whether different people or groups anchor their trust in different sources (see, e.g., Ayalon, 2021; Pagliaro et al., 2021). As we noted above, people seem to express consistent and intercorrelated evaluations of their trust in different actors or institutions, showing a halo of trust across contexts (PytlikZillig et al., 2016). In the present studies, sub‐elements of trust in institutions, in the government COVID‐19 response, and in the prime minister yielded consistent results (see ESM 6). Still, people are able to distinguish between these actors, and so, when focusing on specific places or groups, research aiming to inform policy makers and governmental agents may benefit from distinguishing the exact agents whose trustworthiness is key to foster the public's cooperation (see also Albertson & Gadarian, 2015).

Finally, the cross‐sectional nature of the present studies limits a causal interpretation of the results. In the context of the pandemic, experimentally manipulating trust or concern to inhibit vaccination intention would have been ethically indefensible. However, future research should consider experimental designs to further investigate the interactive effect of concern and trust using analogous but risk‐free scenarios. Concern could be enhanced or decreased by asking participants to recall distressing past events or by exposing them to incidental anxiety‐inducing information (Albertson & Gadarian, 2015). Trust could similarly be enhanced or decreased by presenting manipulated information highlighting the (un)trustworthiness of fictitious political actors. Importantly, such manipulations could isolate the effects of specific facets of trust (e.g., distinguishing between trust, distrust, and mistrust, and/or focusing on specific agents) or dimensions of concern (e.g., self‐concern vs. other‐concern) and advance our understanding of the phenomena. One could also envisage that too high levels of concern can create ironic effects (e.g., denial) if the person feels the threat is too large to address (Lalot et al., 2021).

### Implications for Theory and Practice

Hitherto, trust and concern have generally been addressed in separate theoretical and empirical trenches. The present research provides a strong test of the more general theoretical proposition that it is their combination that is particularly important for capturing the strongest effects of either variable. Prior research showed that low levels of trust result in amplification of the effect of concern about immigration on feelings of outgroup threat (Abrams & Travaglino, 2018). The present evidence shows that low levels of trust accentuate the impact of concern on vaccine hesitancy. These interactive effects suggest interesting avenues for further research because we would expect this principle to apply to other spheres too, such as environmental activism, health behavior, engagement in collective action, and resistance to or acceptance of social change.

Insofar as the present findings generalize, they have significant implications for policy and vaccination campaigns globally. Higher political trust and concern about the pandemic are two factors that can independently motivate positive vaccination intentions, whereas the lack of both is associated with substantially greater hesitancy. This suggests at least two ways to address vaccine hesitancy.

Lack of concern may become a growing problem if the unvaccinated become less concerned once a larger proportion of the population has already been vaccinated. Moreover, because repeated vaccination is required in response to new variants, it may be important to sustain levels of concern that remain above a threshold sufficient to offset any lack of political trust. But strategies to raise levels of concern raise both practical and ethical complexities may require careful targeting specifically those who feel low concern. As briefly mentioned above, there is a risk that invoking even greater concern among those who are already sufficiently concerned could backfire, leading to reactance, denial, or disengagement if the person feels unable to cope (Witte & Allen, 2000). Fear appeals need to be combined with an adequate perception of efficacy—a balance that can be realized but is difficult to achieve in persuasion and information campaigns (Ruiter et al., 2014).

The alternative option is to try to increase political trust. Political trust typically increases at the onset of crises as citizens turn to their leadership for guidance in uncertain times (Hunt et al., 1999). This has been shown in the current crisis as well, with political trust surging in the early months of the pandemic following the implementation of strict governmental rules (e.g., Bol et al., 2021; Davies et al., 2021). However, trust can easily be lost if government action fails to meet citizens' expectations. Measures that are perceived as too strong and impeding people's freedom of choice can also backfire. Recent evidence suggests that the implementation of “health passes” (requiring vaccination to access a wide array of public places) might have increased immediate compliance but did not decrease vaccination hesitancy; in other words, it might have tackled the issue of complacency but not the issue of trust (Ward et al., 2022). Thus, it seems important to avoid antagonizing the vaccine hesitant and the marginalized, and instead to try and regain their trust.

In addition, unethical or rule‐breaking behavior by political leadership can also severely damage political trust (Abrams et al., 2014), as perhaps illustrated by the Dominic Cummings affair in the UK in 2020 (Fancourt et al., 2020) and the prime minister “Partygate” scandal in 2021 (Hayton, 2022). Despite such episodes, however, trust resides at multiple levels (e.g., national, regional, local) and in principle, the presence of a relevant authority that guides by example and maintains a clear and consistent line of communication is more likely to be perceived as efficient and trustworthy (Abrams, Lalot, et al., 2021).

Obviously, vaccine hesitancy relies on trust in actors other than just the government, including doctors and frontline health professionals, pharmaceutical industries and even science in general. Research suggests that communication across the political spectrum is key in such contexts to ensure that politicians and medical experts alike provide a united voice, and clear guidance, to the population (Karafillakis et al., 2022), ultimately encouraging compliance with relevant guidelines, rules and regulations in the longer run—and reducing vaccine hesitancy.

## FUNDING INFORMATION

Research supported by the Nuffield Foundation (grant number WEL/FR‐000022582) and the British Academy (BA Covid and Society, C+S_UoK).

## Supporting information

ESM 1. Study 1: Demographics of the Sample (Breakdown by Nation and Combined Authority)
ESM 2. Timeline of Events in the UK During the Window of Data Collection (December 4, 2020, to March 5, 2021)
ESM 3. Study 1: Additional Analysis Controlling for Conspiracy Beliefs
ESM 4. Participants' Political Orientation and Political Partisanship in Studies 1 and 2
ESM 5. Study 2: Demographics of the Sample: National Data From the UK Census 2021 and YouGov
ESM 6. Iterative Tests of the Political Trust by Concern Interaction in Study 2

## Footnotes

^1^
Anxiety can also lead to greater political trust in the direct aftermath of a crisis (Albertson & Gadarian, 2015) as citizens turn to their leaders for guidance and answers (for accounts of the rally‐round‐the‐flag effect, see also Hetherington & Nelson, 2003; Jennings, 2020). During the pandemic, several papers noted a rapid increase of political trust in the early phase of the pandemic when lockdowns were introduced, but the peak was short‐lived and trust returned to pre‐pandemic levels in a matter of months (e.g., Davies et al., 2021; Kestilä‐Kekkonen et al., 2022). Given that the present studies were conducted over several months in the pandemic, we do not discuss the effect of anxiety on trust further.

^2^
Qualtrics Panels could not offer to recruit sufficient numbers of respondents from many of the small localities that we targeted for this project, making it necessary to reach these respondents through other means, that is, by partnering with local councils.

^3^
Among other scales, the study also included a measure of COVID‐19 conspiracy beliefs. Given that conspiracy beliefs have been found to relate to vaccine hesitancy and, separately, to lower political trust, one might wonder whether the measure might be a better proxy than political trust in the present framework. For the sake of completeness and transparence, we conducted an additional analysis including conspiracy beliefs as a covariate. Results (reported in detail in ESM 3) showed that conspiracy beliefs were significantly and negatively related to vaccination intentions; however, the inclusion of the variable in the model left the Political Trust × Concern interaction term unchanged.

^4^
Political orientation is measured here on the left–right‐wing orientation continuum. Additional information on respondents' political orientation and political partisanship (measured as their self‐reported vote in the most recent general election) is reported in ESM 4 (Studies 1 and 2).
