# Supplementary material for: Distrustful Complacency and the COVID‐19 Vaccine: How Concern and Political Trust Interact to Affect Vaccine Hesitancy
Source: Polit Psychol. 2022 Dec 14:10.1111/pops.12871. Online ahead of print. doi: 10.1111/pops.12871 (PMC9878109; doi:10.1111/pops.12871)
Supplement: Supplementary file 1 — ESM 1. Study 1: Demographics of the Sample (Breakdown by Nation and Combined Authority) ESM 2. Timeline of Events in the UK During the Window of Data Collection (December 4, 2020, to March 5, 2021) ESM 3. Study 1: Additional Analysis Controlling for Conspiracy Beliefs ESM 4. Participants' Political Orientation and Political Partisanship in Studies 1 and 2 ESM 5. Study 2: Demographics of the Sample: National Data From the UK Census 2021 and YouGov ESM 6. Iterative Tests of the Political Trust by Concern Interaction in Study 2 [file POPS-9999-0-s001.docx]

**Electronic Supplementary Material**

**ESM 1. *Study 1:* *Demographics of the sample (breakdown by nation and combined authority)***

|  | GLA | | WECA | | WMCA | | Greater Manchester | |
| --- | --- | --- | --- | --- | --- | --- | --- | --- |
| Demographic categories | Frequ. | % | Frequ. | % | Frequ. | % | Frequ. | % |
| *Gender* |  |  |  |  |  |  |  |  |
| Male | 1186 | 46.3 | 234 | 45.2 | 577 | 44.3 | 499 | 46.9 |
| Female | 1365 | 53.3 | 282 | 54.4 | 719 | 55.2 | 560 | 52.6 |
| Undisclosed | 11 | 0.4 | 2 | 0.4 | 6 | 0.5 | 5 | 0.5 |
| *Age* |  |  |  |  |  |  |  |  |
| 18-24 | 142 | 5.5 | 36 | 6.9 | 102 | 7.8 | 61 | 5.7 |
| 25-34 | 355 | 13.9 | 56 | 10.8 | 156 | 12.0 | 144 | 13.5 |
| 35-44 | 429 | 16.7 | 88 | 17.0 | 230 | 17.7 | 181 | 17.0 |
| 45-54 | 508 | 19.8 | 96 | 18.5 | 246 | 18.9 | 216 | 20.3 |
| 55-64 | 551 | 21.5 | 114 | 22.0 | 287 | 22.0 | 200 | 18.8 |
| 65-74 | 439 | 17.1 | 95 | 18.3 | 213 | 16.4 | 222 | 20.9 |
| 75+ | 138 | 5.4 | 33 | 6.4 | 68 | 5.2 | 40 | 3.8 |
| *Ethnicity* |  |  |  |  |  |  |  |  |
| White / White British | 1878 | 73.3 | 478 | 92.3 | 1010 | 77.6 | 943 | 88.6 |
| Asian / Asian British | 283 | 11.0 | 13 | 2.5 | 149 | 11.4 | 56 | 5.3 |
| Black / African / Caribbean / Black British | 235 | 9.2 | 12 | 2.3 | 73 | 5.6 | 28 | 2.6 |
| Mixed / Multiple ethnicity | 62 | 2.4 | 8 | 1.5 | 25 | 1.9 | 17 | 1.6 |
| Other ethnicity | 44 | 1.7 | 4 | 0.8 | 11 | 0.8 | 6 | 0.6 |
| Undisclosed | 60 | 2.3 | 3 | 0.6 | 34 | 2.6 | 14 | 1.3 |
| *Socioeconomic status* |  |  |  |  |  |  |  |  |
| 8-rung Status ladder: *M* (*SD*) | 4.61 (1.32) | | 4.51 (1.22) | | 4.25 (1.37) | | 4.31 (1.25) | |
| *Political orientation* |  |  |  |  |  |  |  |  |
| Left-wing | 1067 | 41.6 | 184 | 35.5 | 366 | 28.1 | 405 | 38.1 |
| Centre | 810 | 31.6 | 166 | 32.0 | 525 | 40.3 | 369 | 34.7 |
| Right-wing | 681 | 26.6 | 166 | 32.0 | 408 | 31.3 | 288 | 27.1 |
| Undisclosed | 4 | 0.2 | 2 | 0.4 | 3 | 0.2 | 2 | 0.2 |
| Total | 2562 |  | 518 |  | 1302 |  | 1064 |  |

*Note*. The research project encompassed 13 different places in the UK, namely: the nation of Scotland, the nation of Wales, and in England the county of Kent, the metropolitan areas of Greater London, Greater Manchester, West of England Combined Authority (including the cities of Bristol and Bath), and West Midlands Combined Authority (including the city of Birmingham), as well as six smaller local authorities whom councils were partnering with the research team.

*GLA*: Greater London Area, *WECA*: West of England Combined Authority, *WMCA*: West Midlands Combined Authority.

*Continued on next page…*

| …*continued* | Kent | | Other English | | Scotland | | Wales | |
| --- | --- | --- | --- | --- | --- | --- | --- | --- |
| Demographic categories | Frequ. | % | Frequ. | % | Frequ. | % | Frequ. | % |
| *Gender* |  |  |  |  |  |  |  |  |
| Male | 223 | 40.6 | 598 | 38.4 | 271 | 50.7 | 226 | 41.8 |
| Female | 323 | 58.8 | 944 | 60.6 | 261 | 48.8 | 308 | 56.9 |
| Undisclosed | 3 | 0.5 | 16 | 1.0 | 3 | 0.6 | 7 | 1.3 |
| *Age* |  |  |  |  |  |  |  |  |
| 18-24 | 72 | 13.1 | 216 | 13.9 | 52 | 9.7 | 87 | 16.1 |
| 25-34 | 123 | 22.4 | 378 | 24.3 | 138 | 25.8 | 136 | 25.1 |
| 35-44 | 87 | 15.8 | 323 | 20.7 | 139 | 26.0 | 94 | 17.4 |
| 45-54 | 95 | 17.3 | 209 | 13.4 | 86 | 16.1 | 82 | 15.2 |
| 55-64 | 76 | 13.8 | 231 | 14.8 | 62 | 11.6 | 81 | 15.0 |
| 65-74 | 73 | 13.3 | 161 | 10.3 | 46 | 8.6 | 43 | 7.9 |
| 75+ | 23 | 4.2 | 40 | 2.6 | 12 | 2.2 | 18 | 3.3 |
| *Ethnicity* |  |  |  |  |  |  |  |  |
| White / White British | 436 | 79.4 | 1051 | 67.5 | 448 | 83.7 | 455 | 84.1 |
| Asian / Asian British | 16 | 2.9 | 211 | 13.5 | 18 | 3.4 | 13 | 2.4 |
| Black / African / Caribbean / Black British | 21 | 3.8 | 179 | 11.5 | 13 | 2.4 | 5 | 0.9 |
| Mixed / Multiple ethnicity | 9 | 1.6 | 19 | 1.2 | 4 | 0.7 | 12 | 2.2 |
| Other ethnicity | 2 | 0.4 | 24 | 1.5 | 4 | 0.7 | 3 | 0.6 |
| Undisclosed | 65 | 11.8 | 74 | 4.7 | 48 | 9.0 | 53 | 9.8 |
| *Socioeconomic status* |  |  |  |  |  |  |  |  |
| 8-rung Status ladder: *M* (*SD*) | 4.37 (1.21) | | 4.36 (1.31) | | 4.33 (1.21) | | 4.18 (1.20) | |
| *Political orientation* |  |  |  |  |  |  |  |  |
| Left-wing | 243 | 44.3 | 709 | 45.5 | 281 | 52.5 | 284 | 52.5 |
| Center | 162 | 29.5 | 528 | 33.9 | 166 | 31.0 | 161 | 29.8 |
| Right-wing | 144 | 26.2 | 317 | 20.3 | 86 | 16.1 | 95 | 17.6 |
| Undisclosed | 0 | 0 | 4 | 0.3 | 2 | 0.4 | 1 | 0.2 |
| Total | 549 |  | 1558 |  | 535 |  | 541 |  |

*Notes*. *Subjective socioeconomic status* is measured on a ‘status ladder’ with 8 rungs numbered 1 to 8 (a higher number represents higher status). *Political orientation* is measured on a 7-point scale (1 = Left-wing, 4 = Centre, 7 = Right wing). For the table breakout we considered 1-3 as left-wing, 4 as center, 5-7 as right-wing. In the analyses, however, the variable is kept continuous.

**ESM 2. *Timeline of events in the UK during the window of data collection (from 4 December 2020 to 5 March 2021)***

After a relatively calmer period over the summer and early autumn, COVID cases started surging again in the UK during October, prompting the government to impose a second lockdown of England from 5 November to 2 December. Preliminary results on the efficacy of COVID-19 vaccines were published in November, showing encouraging results for both the Pfizer/BioNTech and the Oxford University vaccines (two of the vaccines for which the UK government had reserved doses). The UK became the first country to officially approve the Pfizer/BioNTech vaccine on 2 December and the rollout of vaccinations began on 8 December. During the second half of December much of the media attention focused on Christmas and what restrictions should be put in place during the festivities. On 16 December, London and neighbouring parts of East of England were put under stricter restrictions (Tier 3) following an increase in case numbers in those areas. On 19 December, it is all of London, South East and East of England that went into strict lockdown (Tier 4), scrapping all Christmas plans for millions of people. The total number of COVID-19 cases in the UK was announced to exceed 2 million. Following the announcement of the emergence of a new COVID-19 variant having originated from the South East, more than 40 countries suspended flights from and to the UK on 20/21 December. More areas in England then entered the Tier 4 restrictions, and a third national lockdown was finally imposed on 4 January 2021. When data collection for Study 1 stopped on 2 February, 10.5 million doses of the vaccine had been distributed in the UK, effectively reaching 19% of the adult population (Ritchie et al., 2021).

From the second half of January, number of new cases started to fall steadily, followed by a rapid decrease in numbers of new hospitalisations and deaths visible from the end of January. The government’s target of vaccinating 15 million people before mid-February was reached on 14 February – a “significant milestone.” On 18 February, a study indicated that COVID-19 infections in England had fallen by two-thirds since January. The government unveiled a four-step plan for ending restrictions in England on 22 February, with no major changes expected to happen before at least the end of March. Cases and deaths were continuing to fall steadily when data collection for Study 2 occurred on 5 March, and 21 million people (or 41% of the adult population) had received their first dose of the vaccine by that time (for a full timeline of events, see e.g., Aspinall, 2021).

**References**

Aspinall, E. (2021). *COVID-19 Timeline*. British Foreign Policy Group. <https://bfpg.co.uk/2020/04/covid-19-timeline/>

Ritchie, H., Ortiz-Ospina, E., Beltekian, D., Mathieu, E., Hasell, J., Macdonald, B., Giattino, C., & Roser, M. (2021, 25.01.2021). *Coronavirus (COVID-19) Vaccinations*. Global Change Data Lab. <https://ourworldindata.org/covid-vaccinations>

**ESM 3. *Study 1: Additional analysis controlling for conspiracy beliefs***

Given that conspiracy beliefs have been found to relate to vaccine hesitancy and, separately, to lower political trust, one might wonder whether the measure might be a better proxy than political trust in the present framework. For the sake of completeness and transparence, we conducted an additional analysis including conspiracy beliefs as a covariate and report the results here.

**Measure**

COVID-19 conspiracy beliefs were measured with a single item adapted from Lantian et al. (2016): “I think that the official version of the Covid-19 pandemic given by the authorities hides the truth” (1 = Completely false, 7 = Completely true; *M* = 3.81, *SD* = 1.79; see also Peitz et al., 2021). The score was negatively related to political trust, *r*(8627) = -.30, *p* < .001, and negatively but weakly related to concern, *r*(8627) = -.08, *p* < .001.

**Results**

Following a similar strategy as for the main analyses, we conducted a 3-level multilevel analyses and regressed vaccine hesitancy on political trust, concern, their interaction, and conspiracy beliefs (Step 1). In Step 2, we added demographics as additional covariates (see Table on next page). Results revealed a main effect of conspiracy beliefs, which were negatively related to vaccination intentions. However, the political trust × concern interaction term remained significant despite the inclusion of this covariate, confirming the effect exists above and beyond conspiracy beliefs.

**References**

Lantian, A., Muller, D., Nurra, C., & Douglas, K. M. (2016). Measuring belief in conspiracy theories: Validation of a French and English single-item scale. *International Review of Social Psychology, 29*(1), 1-14. <https://doi.org/10.5334/irsp.8>

Peitz, L., Lalot, F., Douglas, K., Sutton, R., & Abrams, D. (2021). COVID-19 conspiracy theories and compliance with governmental restrictions: The mediating roles of anger, anxiety, and hope. *Journal of Pacific Rim Psychology, 15*, 18344909211046646. <https://doi.org/10.1177/18344909211046646>

***Study 1: Additional analysis controlling for conspiracy beliefs***

|  | Step 1 | | | | Step 2 | | | |
| --- | --- | --- | --- | --- | --- | --- | --- | --- |
|  | *b* (*SE*) | 95% CI | *t*-test | *p*-value | *b* (*SE*) | 95% CI | *t*-test | *p*-value |
| *Constant* | 4.07 (.032) | [4.01, 4.13] | 127.84 | < .001 | 3.97 (.027) | [3.91, 4.02] | 148.57 | < .001 |
| Concern | 0.23 (.013) | [0.21, 0.26] | 18.66 | < .001 | 0.24 (.012) | [0.21, 0.26] | 19.19 | < .001 |
| Political trust | 0.20 (.013) | [0.18, 0.23] | 15.81 | < .001 | 0.14 (.013) | [0.12, 0.17] | 10.84 | < .001 |
| Conspiracy beliefs | -0.34 (.013) | [-0.37, -0.32] | -26.46 | < .001 | -0.29 (.123) | [-0.31, -0.26] | -22.19 | < .001 |
| Concern × trust | -0.10 (.012) | [-0.13, -0.08] | -8.95 | < .001 | -0.10 (.011) | [-0.12, -0.08] | -8.75 | < .001 |
| Gender |  |  |  |  | -0.10 (.012) | [-0.12, -0.08] | -8.02 | < .001 |
| Age |  |  |  |  | 0.20 (.014) | [0.17, 0.23] | 14.22 | < .001 |
| Ethnicity |  |  |  |  | 0.24 (.017) | [0.21, 0.27] | 14.21 | < .001 |
| Socioeconomic status |  |  |  |  | 0.09 (.013) | [0.07, 0.12] | 7.52 | < .001 |
| Political orientation |  |  |  |  | -0.10 (.013) | [-0.12, -0.07] | -7.35 | < .001 |

**ESM 4. *Participants’ political orientation and political partisanship in Studies 1 and 2***

Political orientation plays a minor role in the present findings, but we recognize it can provide interesting information to better understand the sample. In this document we present additional information on participants’ political orientation and partisanship, and the relationship between the two measures.

**Measure**

In both studies, we measured political orientation on a left-right continuum (“When you think of your own political attitudes, where would you place yourself?”, 1 = Left, 4 = Centre, 7 = Right). We also assess partisanship, asking about past vote (“The most recent General Election was held on the 12th of December 2019. Which political party did you vote for, if any?”, 9 parties plus the options to say “other”, “I am not sure”, “I did not vote”, “I was not eligible to vote”, and “I am not registered to vote”).

**Study 1**

**Political orientation across places**

For the sake of this analysis, we considered the two ‘boost’ samples (community activists, and Black & Muslim respondents) separately from the other samples based on geographical location. In contrast, in ESM2, we only focused on geographical area. As could be expected, different samples revealed varying levels of average political orientation, which were aligned with geopolitics.

***Mean scores of political orientation across samples in Study 1***

| Sample | *n* | Political orientation  *M* (*SD*) |
| --- | --- | --- |
| GLA | 2073 | 3.82 (1.44) |
| WECA | 505 | 3.90 (1.42) |
| WMCA | 1017 | 4.07 (1.38) |
| Greater Manchester | 1003 | 3.78 (1.43) |
| Kent | 520 | 3.60 (1.49) |
| Other English | 1285 | 3.53 (1.42) |
| Scotland | 504 | 3.25 (1.38) |
| Wales | 514 | 3.33 (1.40) |
| Community activists | 582 | 3.51 (1.65) |
| Black & Muslim | 626 | 3.19 (1.30) |

**Political orientation and partisanship**

Second, we looked at political orientation as a function of political partisanship (past vote). Scores were aligned with the political agenda of the parties. The more left-wing participants, on average, were those who voted for the Scottish National Party and Labour, followed by Plaid Cymru and Green Party. Liberal Democrats voters were center-leaning-left. Independent, Brexit Party, and Independent Group for Change were more right-leaning. Finally, Conservative voters were the most right-leaning respondents.

Interestingly, left-wing voters seemed to position themselves further on the left than right-wing voters position themselves on the right. Comparing voters of the two main parties, Labour (left) and Conservative (right), scores show a difference of 1.30 with the scale mid-point for Labour against 0.82 for Conservative. This global shift to the left might explain why the average political orientation of the sample leans to the left (*M* = 3.66, *SD* = 1.44) despite a good representation of both main parties.

**
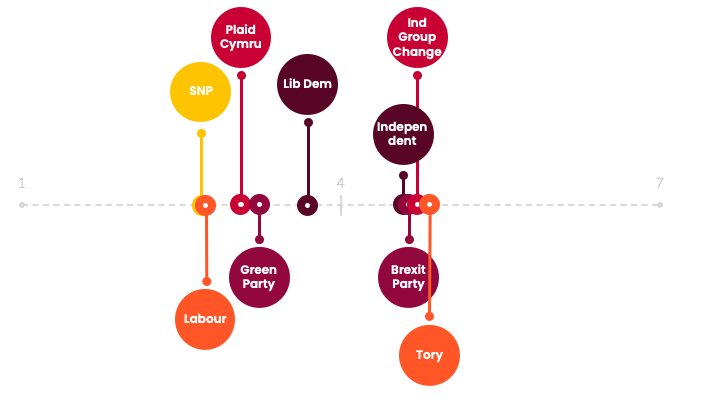
**

***Mean scores of political orientation by past party vote in Study 1***

| Vote | *n* | Political orientation  *M* (*SD*) |
| --- | --- | --- |
| Scottish National Party | 214 | 2.69 (1.31) |
| Labour | 2881 | 2.70 (1.19) |
| Plaid Cymru | 39 | 3.05 (1.17) |
| Green Party of England and Wales | 283 | 3.23 (1.17) |
| Other | 41 | 3.32 (1.42) |
| Liberal Democrats | 814 | 3.69 (1.16) |
| Independent | 45 | 4.58 (1.48) |
| Brexit Party | 144 | 4.64 (1.28) |
| The Independent Group for Change | 14 | 4.71 (1.49) |
| Conservative | 2481 | 4.82 (1.10) |

**Study 2**

**Political orientation and partisanship**

Similar results arise in Study 2. Comparing voters of the two main parties, Labour (left) and Conservative (right), scores show a difference of 1.33 with the scale mid-point for Labour against 0.88 for Conservative.

***Mean scores of political orientation by past party vote in Study 2***

| Vote | *n* | Political orientation  *M* (*SD*) |
| --- | --- | --- |
| Other | 5 | 2.60 (0.55) |
| Scottish National Party | 39 | 2.67 (0.96) |
| Labour | 353 | 2.67 (1.05) |
| Plaid Cymru | 5 | 2.80 (0.84) |
| The Independent Group for Change | 1 | 3.00 (-) |
| Green Party of England and Wales | 43 | 3.28 (0.96) |
| Liberal Democrats | 100 | 3.45 (1.05) |
| Brexit Party | 12 | 4.50 (1.38) |
| Conservative | 318 | 4.88 (1.07) |
| Independent | 3 | 5.67 (1.53) |

**ESM 5. *Study 2:* *Demographics of the sample. National data from the UK Census 2021 and from YouGov are reported for comparison purposes***

|  | Study 2 sample | | UK Census 2021 |
| --- | --- | --- | --- |
| Demographic categories | Frequ. | % | % |
| *Gender* |  |  |  |
| Male | 520 | 48.8 | 49.0 |
| Female | 542 | 50.8 | 51.0 |
| Undisclosed | 4 | 0.4 | - |
| *Age* |  |  |  |
| 18-24 | 74 | 6.9 | 7.9 |
| 25-34 | 230 | 21.6 | 17.6 |
| 35-44 | 189 | 17.7 | 16.9 |
| 45-54 | 177 | 16.6 | 17.3 |
| 55-64 | 248 | 23.3 | 16.3 |
| 65-74 | 128 | 12.0 | 12.9 |
| 75+ | 20 | 1.9 | 11.2 |
| *Ethnicity* |  |  |  |
| White / White British | 906 | 85.0 | 84.8 |
| Asian / Asian British | 82 | 7.7 | 8.0 |
| Black / African / Caribbean / Black British | 38 | 3.6 | 3.5 |
| Mixed / Multiple ethnicity | 20 | 1.9 | 1.8 |
| Other ethnicity | 15 | 1.4 | 1.9 |
| Undisclosed | 5 | 0.5 | - |
| *Socioeconomic status* |  |  |  |
| 8-rung Status ladder: *M* (*SD*) | 4.62 (1.18) | | - |
| *Political orientation* |  |  | YouGov (2021) |
| Left-wing | 483 | 45.3 | 37 |
| Center | 312 | 29.3 | 30 |
| Right-wing | 271 | 25.4 | 33 |
| Total | 1066 | 100% | 100% |

*Notes*. *Subjective socioeconomic status* is measured on a ‘status ladder’ with 8 rungs numbered 1 to 8 (a higher number represents higher status). *Political orientation* is measured on a 7-point scale (1 = Left-wing, 4 = Center, 7 = Right wing). For the table breakout we considered 1-3 as left-wing, 4 as center, 5-7 as right-wing. In the analyses, however, the variable is kept continuous.

*UK Census 2021*: data from 21^st^ March 2021 retrieved from <https://census.gov.uk/>. *YouGov (2021)*: data retrieved from <https://yougov.co.uk/topics/philosophy/trackers/what-political-alignment-is-the-british-public>, using an aggregate of the data from 11^th^ February and 8^th^ April 2021 to match the time period of the census. Percentages are computed while excluding *Don’t know* answers (26%).

**ESM 6. *Iterative tests of the political trust by concern interaction in Study 2***

Study 2 relied on multi-item measures of trust and concern, which allows for supplementary analyses testing the effect of specific subsets of items. In this document we report the results of these iterative analyses.

**Measures**

**Concern**

Concern was measured with 3 items (see Table 3), which we sub-categorized as one self-concern item (“How concerned are you about consequences of the pandemic for you personally (such as your health, financial or other aspects)?”) and two concern for others items (“How concerned are you about consequences of the pandemic for the people in your local area?”, “How concerned are you about consequences of the pandemic for the people in the UK in general?”). We also considered the aggregated index (3 items) as in the main analysis.

**Political trust**

The study had 7 items for political trust, which we sub-categorized into indices of general political trust (3 items: “Politicians are mainly in politics for their own benefit and not for the benefit of the community”, “Most members of the UK Parliament are honest”, “How much trust do you have in the UK government?”), trust in the government’s response to COVID-19 (2 items: “I believe the UK Government is handling the causes and consequences of the pandemic competently”, “How well or badly do you think the UK Government is handling the issue of the coronavirus?”), and trust in Prime Minister Boris Johnson (“Over the next year, how much do you think Boris Johnson (Prime Minister and leader of the Conservative Party) can be trusted to handle the pandemic for the UK as a whole?”, “Over the next year, how much do you think Boris Johnson (Prime Minister and leader of the Conservative Party) can be trusted to handle the pandemic for England?”). We also considered the aggregated index (7 items) as in the main analysis.

**Results**

In the table below we report the test for the interaction term for each combination of 3 indices of concern × 4 indices of trust. Those are extracted from multilevel linear regression models (county/nation) with demographics entered as covariates (i.e., the equivalent of “Step 2” model for the main analysis), using the 2-item vaccine hesitancy index as DV.

As can be seen from the table, results were remarkably consistent. Of the 12 combinations only one was found nonsignificant although descriptively going in the expected direction (self-concern × trust in the government’s response to COVID-19). Thus, these additional results suggest that the interactive effect of trust and concern is robust and does not depend on the specific items used to assess them.

***Statistical tests of the concern × trust interaction term (t-test and associated p-value), considering all combinations of sub-factors of concern and trust***

|  | Self-concern | Concern for others | Aggregated concern (3 items) |
| --- | --- | --- | --- |
| General political trust | ***t* = -2.37, *p* = .018** | ***t* = -3.02, *p* = .003** | ***t* = -3.08, *p* = .002** |
| Trust in COVID response | *t* = -1.61, *p* = .109 | ***t* = -3.23, *p* = .001** | ***t* = -3.03, *p* = .003** |
| Trust in the Prime Minister | ***t* = -2.12, *p* = .034** | ***t* = -2.52, *p* = .012** | ***t* = -2.77, *p* = .006** |
| Aggregated trust  (6 items) | ***t* = -2.55, *p* = .011** | ***t* = -3.19, *p* = .001** | ***t* = -3.35, *p* < .001** |

*Note*. Significant findings appear in bold.
